# Synovial mast cells from knee and hip osteoarthritis: histological study and clinical correlations

**DOI:** 10.1186/s40634-022-00446-2

**Published:** 2022-01-25

**Authors:** L. Farinelli, A. Aquili, M. Mattioli-Belmonte, S. Manzotti, F. D’Angelo, C. Ciccullo, A. Gigante

**Affiliations:** 1 Clinical Orthopaedics, Department of Clinical and Molecular Science DISCLIMO, Università Politecnica Delle Marche, Ancona, Italy; 2 Department of Clinical and Molecular Science DISCLIMO, Università Politecnica Delle Marche, Ancona, Italy

**Keywords:** Osteoarthritis, Synovial mast cells, Synovium, Inflammation, Level of evidence, Basic science study

## Abstract

**Purpose:**

The aim of this study was to investigate the presence of synovial mast cells (MCs) in hip and knee tissue from osteoarthritis (OA) patients and to correlate them with clinical and radiological data.

**Methods:**

Synovial tissue was obtained during arthroplasty from 60 patients, 30 with knee OA and 30 with hip OA. Control synovial tissue was obtained from 30 patients without OA, 15 undergoing above-knee amputation and 15 receiving a hip replacement for fracture. Before surgery, the radiographic findings were graded according to the Kellgren-Lawrence system and clinical data including pain (VAS) and functional information (KOOS and HOOS) was collected. The tissue was stained with hematoxylin–eosin and toluidine blue for histochemistry and incubated with CD117 and CD31 antibodies for immunohistochemistry. MC and vessel number and synovitis score were determined in all samples.

**Results:**

Mean MC number, synovitis score and vessel number were significantly higher in the OA samples (*p* < 0.05) than in control tissue. MC number correlated with the synovitis score and disease severity in both patient groups.

**Conclusions:**

The prevalence of MCs in synovium from OA patients and their association with synovial inflammation and pain suggest a role for them in OA pathophysiology.

## Introduction

Osteoarthritis (OA) is characterized by cellular stress and extracellular matrix degradation. These processes are initiated by injury, which activates maladaptive repair responses, including innate immunity pro-inflammatory pathways. The disease is characterized by cartilage breakdown, osteophyte formation, subchondral bone sclerosis, alterations of the joint capsule, and synovial inflammation [15]. Despite the latter feature, OA is not usually classified as an inflammatory condition, since the leukocyte count in synovial fluid is typically below the threshold for “inflammatory disorders” [10]. However, clinical symptoms such as palpable joint swelling, night pain, and morning stiffness are strong indicators of synovitis. Furthermore, inflamed synovium at the border of early cartilage lesions [2] and the association between substantial synovial inflammation and rapidly destructive OA strongly indicate that synovitis plays a major role in OA pathogenesis and progression [18].

Inflammation of the synovial membrane is found in early as well as late OA. Immune cells infiltrating the synovial tissue may be important determinants of synovial inflammation [19]. The predominant types of immune cells found in OA are macrophages, T lymphocytes and mast cells (MCs).

MCs are sentinels of the innate immune system that provide a rapid response to exogenous pathogens and endogenous danger signals [4]. A broad variety of factors that stimulate FceRI or toll-like receptor (TLR) on the MC surface can influence MC degranulation and induce the release of pre-formed mediators such as cytokines, chemokines, histamine, tryptases and pro-inflammatory lipids, leading to synovial inflammation, angiogenesis and bone destruction. It has been suggested that bone or cartilage breakdown products acting as antigens may stimulate MC degranulation [17, 20]. It has also been reported that infiltrating immune cells and cytokine expression are higher in rheumatoid arthritis than in OA; however, the different MC enrichment may reflect a qualitatively different inflammation in the two conditions [8]. Notably, MC activation and degranulation via the IgE/FceRI/Syk axis has been reported to mediate inflammation and tissue damage in a mouse model of OA, whereas inhibition of FceRI signaling reduced OA severity [6, 21]. Moreover, a fairly recent investigation into the effect of anti-IgE therapy on atopic patients with knee OA found significantly reduced pain (EQ VAS) and disability (KOOS) during follow-up, suggesting that the symptom improvement was due to the partial blockage of IgE-related MC activation [1].

Despite their relevance, MCs have not been extensively investigated and their role in synovial inflammation and OA pathophysiology is poorly understood. Crucially, a limited number of studies have addressed the correlation between MC number, degree of synovial inflammation and clinical parameters (i.e., pain and radiological progression) in knee and hip OA [9, 11].

The aim of this study was to gain insight into the role of MCs in OA. We first evaluated MC number and degree of inflammation in knee and hip synovial tissue from OA patients; we then correlated MC number with patients’ preoperative pain and functional test scores, to establish whether MC number correlated with the radiological grade of OA. For comparison, we analyzed synovial tissue from the knee and hip joints of patients without OA. The null hypothesis was a significant difference between MC number and synovial inflammation between OA patients and control subjects.

## Materials and methods

### OA patients

Synovial tissue was obtained during arthroplasty from patients with knee (*n* = 30) or hip (*n* = 30) OA treated at Azienda Ospedaliera-Universitaria Ospedali Riuniti of Ancona (Ancona, Italy) from January 2018 to January 2021. Patients provided their written informed consent to participate in the study, whose inclusion and exclusion criteria are summarized in Table 1.

**Table 1:** Inclusion and exclusion criteria of patients with knee and hip osteoarthritis

Inclusion criteria	Exclusion criteria
Age > 50	Cardiovascular or neurological disorders
History of chronic pain (> 4 months)	Diabetes
Limitation of daily activities,	Atopy
Radiographic findings of degenerative joint changes (Kellgren-Lawrence grade III-IV)	Immunosuppressants or hip injections in the previous 6 months
	Other rheumatic diseases
	Knee/hip surgery
	Osteonecrosis
	Perthes disease, dysplasia, epiphysiolysis

### Control group

Knee synovial tissue was obtained from 15 patients without OA, 8 men and 7 women whose mean age was 53.2 years (range 45 – 67), who underwent above-knee amputation. None of these patients had a history of knee OA, arthralgia/arthritis or knee surgery. Amputation was due to vascular disease (*n* = 7), high-energy lower limb trauma (*n* = 6), or neoplasm (*n* = 2).

Hip synovial tissue was obtained from 15 patients without OA, 4 men and 11 women with a mean age of 65.4 years (range 55–78), who underwent arthroplasty for medial femoral neck fracture. None of these patients had a history of hip OA, arthralgia/arthritis or hip surgery.

### Clinical and radiological data

Before surgery, self-reported pain was assessed using the visual analog scale (VAS, 0–100), and the Hip Disability and Osteoarthritis Outcome Score (HOOS) and the Knee Injury Osteoarthritis Outcome Score (KOOS) were calculated. Standardized radiographs (fixed flexion posteroanterior for the knee and anteroposterior of the pelvis for the hip) were obtained from participants and graded according to the Kellgren-Lawrence (KL) classification by an experienced musculoskeletal radiologist who was blind to patient characteristics.

### Histological and histomorphometric analysis

Synovial tissue collected during surgery was fixed in 10% buffered formalin, embedded in paraffin and sectioned to a thickness of 3–5 µm. Sections were dewaxed in xylene and rehydrated through a graded ethanol series (Bio-Optica SpA, Milano, Italy). For routine histological examination, sections were stained with hematoxylin–eosin and toluidine blue metachromatic staining. Polysine® slides (Menzel-Gläser, Braunschweig, Germany) were used for immunohistochemistry. Endogenous peroxidase activity was quenched by incubating the sections in 3% H_2_O_2_ for 10 min at room temperature. Subsequently, sections were incubated with polyclonal rabbit anti-human CD117 and with monoclonal mouse anti-human CD31 antibody, diluted respectively 1:50 and 1:40 in EnVision FLEX Antibody Diluent (Dako Agilent Technologies, Carpinteria, CA, USA), in a humidified atmosphere for 20 min. The antigen–antibody complex was detected using the Dako EnVision™ + Dual Link System, HRP/DAB, according to the manufacturer's protocol (Dako Agilent Technologies, Carpinteria, CA, USA). Sections were counterstained with Mayer's hematoxylin (Bio-Optica SpA). Sections processed without the primary antibody were the negative controls.

Sections were examined with a Leica Leitz DMRBE (Leica Microsystem, Wetzlar, Germany) light microscope equipped with a digital analyzer. Each area of the tissue sections was photographed and digitalized using an image processing system (LAS and Qwin Plus, Leica Inc., Wetzlar Germany). MCs were detected with toluidine blue. Since this stain may be less sensitive than tryptase [8], sections were incubated with CD117 antibody to detect partially as well as totally degranulated MCs.

The total number of MCs (degranulated and not degranulated) and of vessels was determined in 10 adjacent high-power (40 × magnification) fields (hpf) in the center of the section, starting on the left side at the lining layer. In the same sections, the area fractions (%) occupied by MCs and vessels were evaluated with the image processing system in 10 fields/specimen at 20 × magnification.

CD31 immunolabeling allowed identifying blood vessels as areas surrounded by positively stained endothelial cells. Vessel area was measured including the perimeter and lumen. Two independent observers, blinded to sample origin and characteristics, counted the MCs and vessels using the image processing system and checked its results manually.

Data are expressed as mean ± standard deviation (SD). To assess inflammation, samples were scored according to Krenn et al. [13] for the enlargement of the lining cell layer, the cell density of the synovial stroma and the inflammatory infiltrate. The changes were ranked on a scale from none (0) to slight (1), moderate (2), and strong (3). The values of the parameters were combined according to the synovitis score as follows: 0–1, no synovitis; 2–4, low-grade synovitis; and 5–9, high-grade synovitis [13]. The inflammatory infiltrate was examined at 10 × magnification, whereas the synovial lining layer and synovial stroma cell density were viewed at 40 × magnification.

### Statistical analysis

Statistical analysis was performed using the SAS statistical package (Statistical Analysis System Institute). Results are expressed as mean ± SD. ANOVA and Student's t-test were used to analyze the differences between groups. Correlation studies were performed by linear regression analysis, using Pearson’s correlation coefficient ρ. Significance was set at *p* < 0.05 (95% confidence interval).

## Results

The clinical data of all participants are summarized in Table 2.

**Table 2:** Clinical data of the 60 osteoarthritis patients and 30 control subjects

	Knee		Hip	
	OA	Control	OA	Control
Number	30	15	30	15
Age (years), mean (SD)	73.18 (7.7)	53.2 (8.17)	69.8 (6.3)	65.4 (7.2)
Female, no. (%)	11 (36.67%)	7 (47%)	19 (63.33%)	11 (73%)
KL 3 no. (%)	13 (43.3%)		18 (60%)	
KL 4 no. (%)	17 (56.7%)		12 (40%)	
Pain VAS, mean (SD) [95%CI]	78.5 (10.27) [74.67–82.33]		78.67 (6.94) [76.08–81.26]	
KOOS/HOOS, mean (SD) [95%CI]	31.98 (7.73) [29.09–34.87]		31.41 (7.37) [28.66–34.16]	

*OA* = osteoarthritis, *VAS* = Visual analog scale, *KL* = Kellgren-Lawrence system, *KOOS* = Knee Injury and Osteoarthritis Outcome score, *HOOS* = Hip Disability and Osteoarthritis Outcome Score, *SD* = standard deviation, *CI* = confidence interval

The number of MCs and the area fraction occupied by these cells were significantly higher in knee and hip OA patients than in control subjects (*p* < 0.05) (Table 3*)*. As expected, the synovitis score was also higher in these patients (*p* < 0.05). Notably, MC number and the synovitis score were significantly higher in knee OA than in hip OA patients (*p* < 0.05). Vessel number and area fraction were higher in the OA patients than in controls (*p* < 0.05).

**Table 3:** Evaluation of the mast cell (MC) infiltrate and Synovitis score in the 60 osteoarthritis (OA) patients. SD: standard deviation; * significant differences with *p* < 0.05 in Knee and Hip groups

	Knee		Hip	
	OA	Control	OA	Control
MCs /field (SD) [CI] *	165.1 (76.88) [136.36–193.78]	20 (5.92) [5.3–34.7]	107.77 (23.55) [94.73–120.81]	14 (2.74) [10.6–17.4]
Area fraction (SD) (%) *	0.65 (0.4)	0.10 (0.09)	0.31 (0.12)	0.04 (0.02)
Lining layer (SD) *	1.58 (0.79)	0.6 (0.42)	1.2 (0.73)	0.5 (0.45)
Stromal activation (SD) *	1.5 (0.52)	0.2 (0.51)	1.3 (0.62)	0.4 (0.55)
Infiltrate (SD) *	1.41 (0.52)	0.3 (0.43)	1.27 (0.45)	0.2 (0.45)
Synovitis score (SD) *	3.77 (1.55)	0.4 (0.55)	2.8 (0.92)	0.6 (0.55)
Vessel no. mean (SD), [IC95%] *	59.48 (19) [52.25–66.71]	25.2 (3.11) [21.33–29.07]	54.44 (14.11) [47.42–61.46]	16.4 (6.23) [8.67–24.13]
Vessel area fraction (SD) (%) *	6.3 (1.8)	0.8 (0.3)	3 (0.4)	0.6 (0.3)

**Fig. 1:**
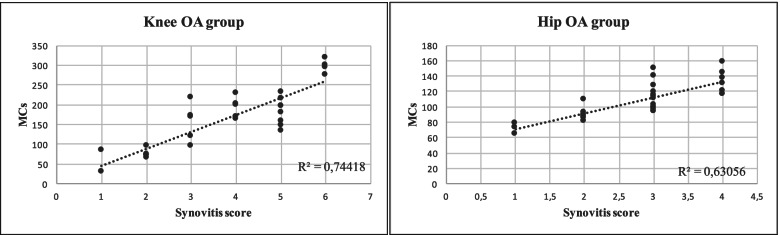
Correlation of synovitis score and synovial mast cell number in knee and hip tissue from patients with osteoarthritis. R2, Pearson’s correlation coefficient

**Fig. 2:**
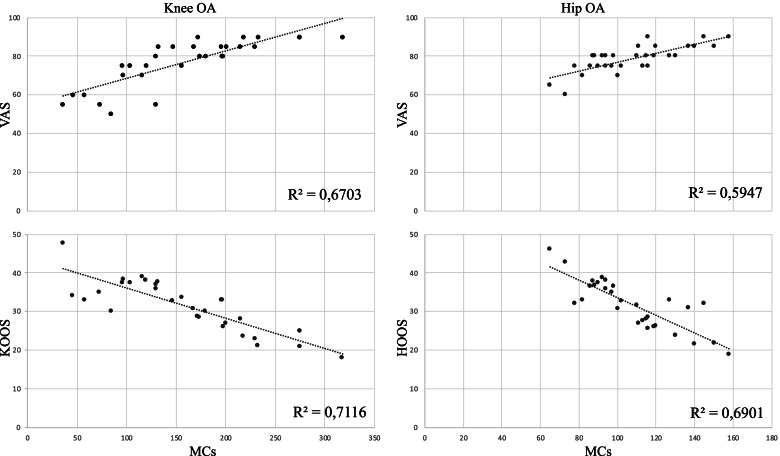
Correlation of synovial mast cell number and functional status of patients with knee or hip osteoarthritis

Investigation of MC number in relation to the degree of synovial inflammation highlighted a positive correlation with the synovitis score in both OA groups (Fig. 1). The knee OA group was also characterized by a higher Pearson’s correlation coefficient (R^2^). Analysis of MC number in relation to the clinical data indicated a positive correlation with the VAS score, stronger in the knee OA group, and a negative correlation with KOOS and HOOS values in both OA groups (Fig. 2*)*.

Finally, the investigation of MC number in relation to the radiographic stage showed that patients with KL grade IV had a higher though non-significant MC number than those with KL grade III.

## Discussion

**Fig. 3:**
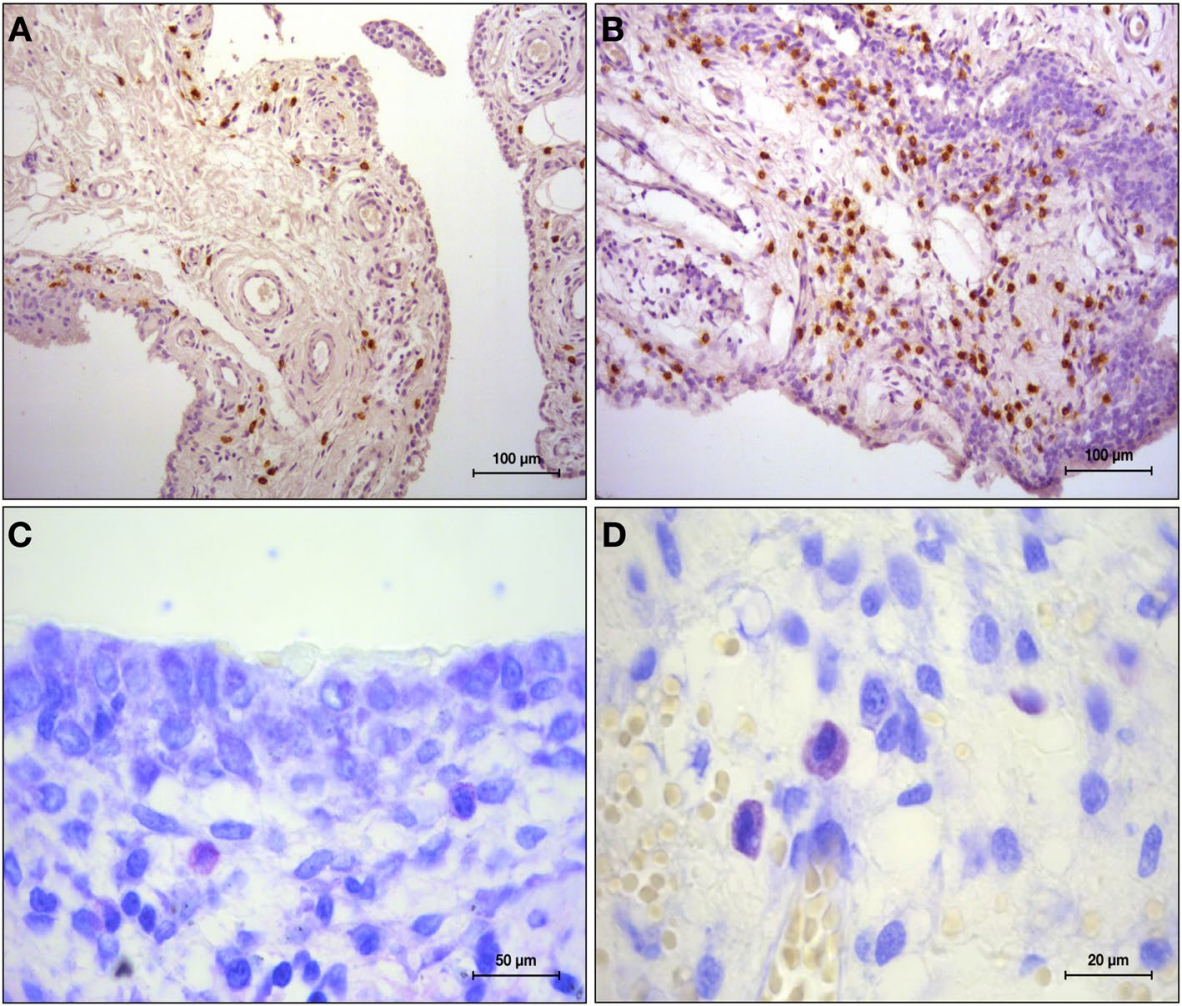
Sample from a patient with knee osteoarthritis. Paraffin sections stained for CD117 (**A**, **B**) and toluidine blue (**C**, **D**). **A** and **B**, 20 × magnification; **C** 40 × magnification; **D** 100 × magnification. In **A** and **B,** mast cells are identified by brown reaction product. In **C** and **D,** mast cell show metachromasia

The [1] finding of the present study was the significantly higher number of MCs counted in the synovium of OA patients compared to controls. MCs were predominantly found in the sub-lining layer in all participants, as reported in previous works [5, 7] (Fig. 3).

**Fig. 4:**
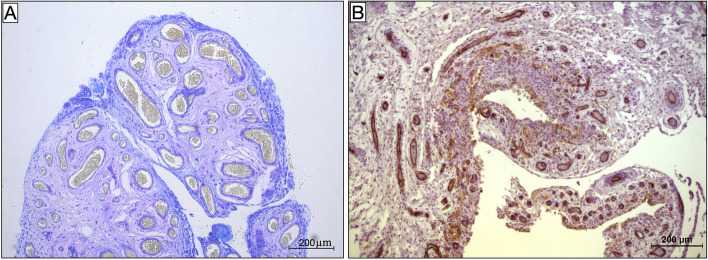
**A, B**. Synovial hypervascularity. **A**. Toluidine blue staining; **B.** Paraffin sections stained for CD31. Both images, 10 × magnification

MCs and their mediators are known to be found in the synovial membrane and fluid of OA patients [14]. Interestingly, our data indicate that a greater MC abundance was associated with more severe synovitis and worse preoperative clinical scores. It has recently been reported that intra-articular injection of cultured MCs induced significant cartilage degeneration, synovial inflammation and pain worsening in an OA mouse model, suggesting that OA flares may be partly due to MC activation [6]. In another study, pharmacological interventions directly targeting MCs or their inflammatory pathways reduced OA severity in a mouse model of OA, since inhibition of IgE-mediated FceRI signaling reduced osteophyte formation [21]. Furthermore, analysis of our data demonstrated that patients with KL grade IV had a higher number of MCs, which suggests a role for them in bone remodeling in OA. In contrast, it is still unclear whether the role of MCs in disease pathogenesis depends on IgE/FceRI signaling. Several studies have suggested that bone or cartilage breakdown products, detected in patients with joint instability and injury, might induce MC activation via TLRs found on the MC surface [3, 12]. Once activated, MCs might promote synovitis by recruiting inflammatory cells, inducing synovial fibroblast hyperplasia and enhancing vessel permeability and angiogenesis through the release of inflammatory mediators such as histamine, proteases, interleukin (IL)-1, IL-6, tumor necrosis factor-alpha (TNF-alfa) and vascular endothelial growth factor (VEGF) [16]. Indeed, our patients exhibited a marked synovial hypervascularity compared to controls (Fig. 4).

**Fig. 5:**
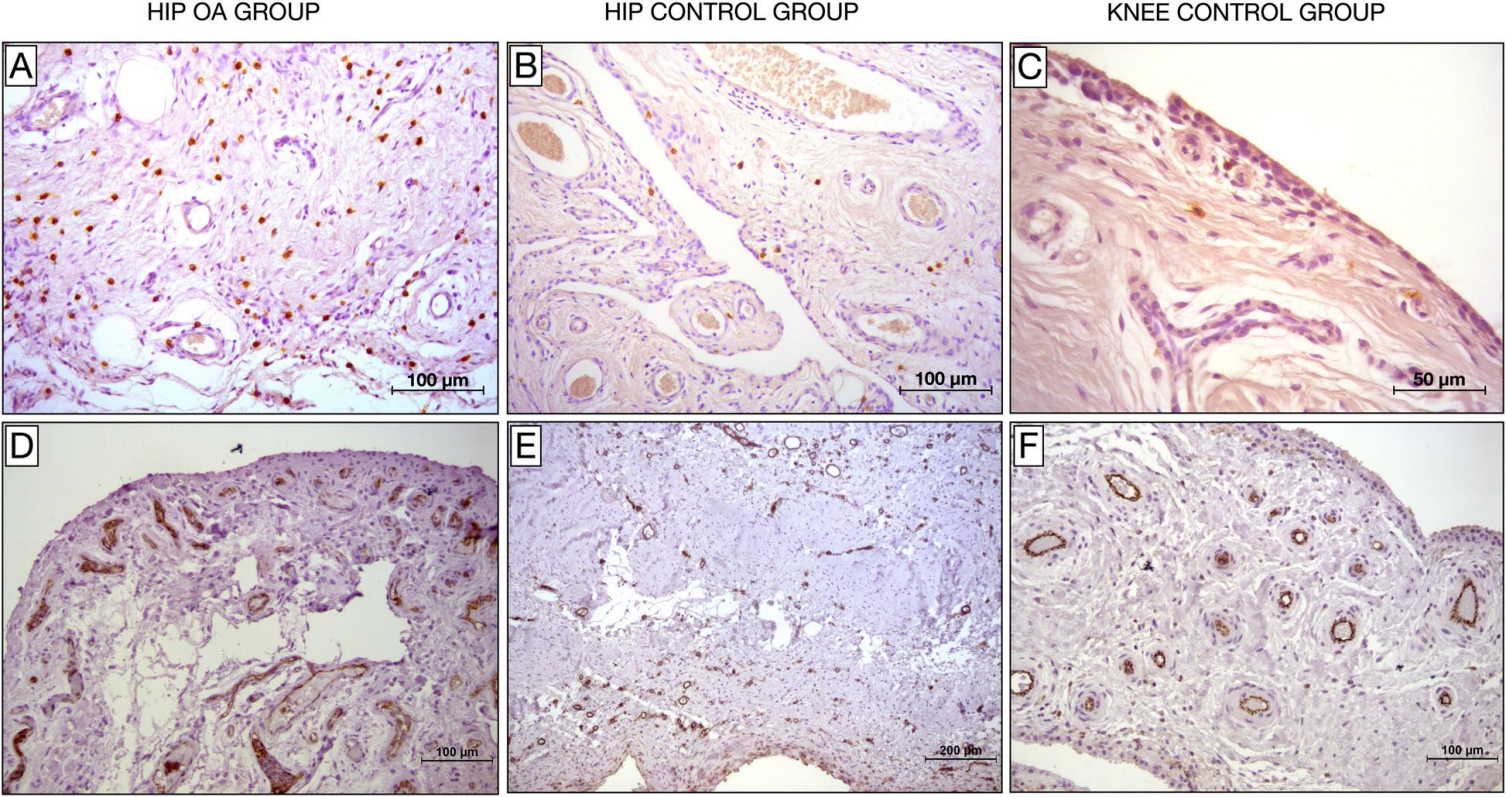
Samples from patients with hip osteoarthritis and from hip and knee control groups. **A, B** and **C**. Paraffin sections stained for CD117. Mast cells are identified by brown reaction product. **B** and **C.** Low grade of synovial inflammation. **A** and **B**, 20 × magnification; **C**, 40 × magnification. **D, E** and **F**. Paraffin sections stained for CD31. Vessels are identified by brown reaction product. Significant synovial hypervascularity can be seen in D

In addition, synovial tissue from hip OA was characterized by a lower grade of inflammation than knee OA tissue (Fig. 5). To date, no studies have been conducted to explore the reason for these differences. It is reasonable to hypothesize that the differences in hip and knee joint anatomy, biomechanics and loading pattern result in different OA pathogenesis and synovial inflammation.

The chief limitations of the study include the facts that we did not establish the role of MCs in synovial inflammation and OA pathogenesis and that our findings come from a small cohort of patients with advanced OA. On the other hand, the inclusion of a control group for each patient group is a clear strength of our work. The control participants had a negative history of pain or joint-related diseases before above-knee amputation or hip replacement and were therefore assumed not to suffer from OA or other diseases that may cause synovial inflammation.

In conclusion, the presence of MCs in the synovium of patients with OA and their association with synovial inflammation and pain suggest a possible role for them in OA pathogenesis. An in vitro OA model is expected to provide insight into the direct effect of MCs on chondrocytes, of cell–cell interactions, and of the mediators that may be involved. Our findings open the way for the investigation of the role of MCs in OA and for new disease-modifying treatments targeting the multiple functions of MCs.
